# Influence of Traffic-Induced Vibrations on Humans and Residential Building—A Case Study

**DOI:** 10.3390/ijerph19095441

**Published:** 2022-04-29

**Authors:** Damian Beben, Tomasz Maleska, Piotr Bobra, Józef Duda, Wojciech Anigacz

**Affiliations:** Faculty of Civil Engineering and Architecture, Opole University of Technology, 45-758 Opole, Poland; t.maleska@po.edu.pl (T.M.); p.bobra@po.edu.pl (P.B.); jo.duda@po.edu.pl (J.D.); w.anigacz@po.edu.pl (W.A.)

**Keywords:** traffic vibration, building damage, perception level of vibrations, sustainable development

## Abstract

The case study presents an assessment of the traffic-induced vibrations on humans and residential buildings, which is important for sustainable development. The analyzed residential building had several cracks in the walls. Control gypsum tapes were applied to all cracks in the building and additional elements near the road to determine the propagation of the damage. To determine the harmfulness of vibrations for humans, vibration acceleration measurements linked to road traffic inside the analyzed building were carried out. The vibration velocities inside the object were set based on the integration of the obtained accelerations. The experimental field investigation was carried out in places where humans commonly stayed (on the first floor) at the points where the vibrations are transmitted from the construction to humans. The study involved a time history analysis, a Fast Fourier Transform (FFT) analysis, and Root Mean Square (RMS) acceleration and velocity in a one-third octave bands spectrum. Based on the conducted experimental tests, it can be pointed out that the received velocity values in the tested building, caused by the passage of various vehicles, were below the permissible levels. However, it was noticed that the distance between the building and the fence had an important role in damping vibrations emitted by passing vehicles. The presented case study may be of use to other researchers who will be involved in similar cases and want to include sustainable infrastructure development.

## 1. Introduction

The increasing road and rail traffic has a harmful influence on the technical state of buildings and the quality of life of the inhabitants [1,2], as well as on sustainable development. The discussed problem is quite complicated because it is related to various fields such as the dynamics of structures, propagation of waves in soil, acoustics and individual human reaction to psychophysical stimuli [3]. Therefore, in recent years, an increase in this area of research has been observed. This problem was analyzed in particular in the field of vibrations caused by passing vehicles, i.e., cars [4,5,6] and trains [7,8,9,10,11,12,13,14,15,16,17,18,19].

In the area of vibration impact analysis on buildings, the main goal was to examine the influence of the motorway-induced vibration level on the resulting damage to the structure. In this aspect, interesting results of experimental research with the use of ambient noise were presented in the paper [20]. In this case, the determination of the stiffness of the building is able to determine the impact of the recorded vibrations on the building. Measurement and analytical procedures for buildings with irregular shapes and a relatively simple shape (residential building) were presented. For experimental research, the two lowest frequencies (10.5 Hz and 11.9 Hz) measured during the measurements were used. These studies were carried out in addition to previous work [21,22]. On the other hand, the paper [23] investigated the influence of road surface roughness during heavy vehicle passage. The Authors found that the type of road surface affects the wave propagation and the quality of life near the road. In this paper, a heritage building in Naples was analyzed. The measurements were related to vehicle type and speed and were compared with values obtained by our own model. In turn, in the work [24], a numerical analysis of vibration reduction was carried out by using a reinforced concrete slab located on the ground. In this work, guidelines were developed for the design of new buildings using a reinforced concrete damping slab. It should be emphasized that the FEM model was calibrated on the basis of experimental studies.

Nowadays, more and more research concerns the so-called structural health monitoring (SHM), where, for example, with the use of the analysis of structural vibrations, its rigidity is determined. As a result, it is possible to locate damage by propagating vibrations in the structure. In addition, one can determine the size and place of damage in real time. On this aspect, research was also carried out in [25,26,27,28,29]. Such monitoring is typically used in buildings exposed to strong dynamic impacts and/or in buildings where significant structural damage has already occurred.

A big challenge for scientists and engineers is to analyze the data obtained from the measurements. A common practice in scientific works is the use of filters (noise leveling, one-third octave band spectrum analysis) to emphasize particular effects of vibration [30,31,32,33,34,35,36]. It should be added that almost every case requires an individual approach due to somewhat various soil and environmental conditions, and above all, various types of buildings and a different source of vibrations. Hence, the best manner to assess the dynamic influence on a building and/or humans is to directly measure acceleration, vibration frequency and rotation [37,38,39].

Low-intensity para-seismic events (mining tremors) can also generate a similar impact on humans as vibrations from road traffic. Research on this topic of residential and office buildings was presented by, among others, [40,41,42]. In these works, the permissible norms for vibrations of low intensity (comparable to road vibrations) were not exceeded, but the vibrations were so burdensome that they interfered with the living conditions of residents. The influence of road surface on vibration intensity is shown in [43]. In addition, the influence of anthropogenic vibration on civil engineering objects in comparison with vibrations recorded on the ground and the foundations is described in [44,45], where the lower limit of perceptibility of vibrations by the building and the lower limit of the dynamic impact were not exceeded. Minimization of the impact of vibration and vibration annoyance is analyzed in [45].

The main aim of our case study was to determine the impact of vibrations caused by passing vehicles on a residential building and the consequent living conditions for people. The research was carried out on an existing single-family residential building. Based on the available literature and standards, the results of acceleration and velocity in relation to the permissible standard values were analyzed. The research carried out in this paper shows a very important problem of road traffic, through which the inhabitants of the building cannot live freely, even though the permissible standards for vibrations have not been exceeded.

The authors distinguish two novelties in this study. The first concerns the subject of the research, i.e., despite the cracks in the old building noticed by the residents and nuisance vibrations, the permissible standards were not exceeded. It is also interesting that the old building responds well to increased vehicle traffic.

The second novelty concerns the applied test method, i.e., implementation-unified methodology containing RMS analysis in a one-third octave bands spectrum for both acceleration and velocity. This methodology allows to accurately determine the frequencies causing noticeable discomfort in humans, by contravening the permissible vibration levels in residential buildings in accordance with international standards. As described in [46], this methodology can be implemented for other vibration sources but has not yet been verified for road-induced vibration. Previously this method was used, among others for railway-induced vibration analysis. Therefore, in this case study, it was decided to use the above-mentioned method to analyze road-induced vibration.

The results obtained from the study may be useful for the assessment of sustainable development, including planning the construction of new roads and industrial plants in the vicinity of human habitats.

## 2. Methodology Description of Research

### 2.1. Description of the Building and the Occurred Cracks

**Figure 1:**
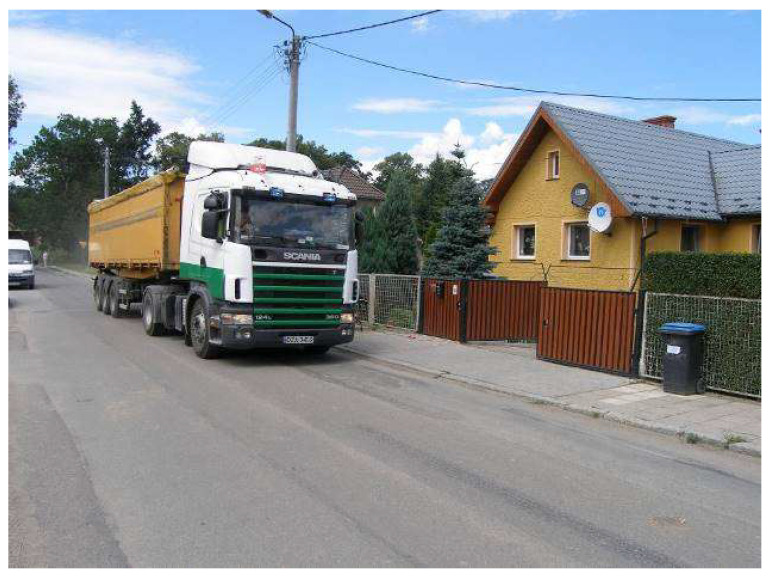
The analyzed building during measurement of vibrations.

The analyzed residential building was constructed before World War II and is situated by a country road. The building is situated in accordance with the current regulations [47] and is at the correct distance from the road. The walls of the building are 0.65 m thick, and are made of brick. The structural ceiling is executed of wood. It is a two-story house without a basement. In the 1990s, the building underwent a renovation, increasing its functioning. The building is located at a distance of 12.0 m from the road axis. Figure 1 presents the front view of the analyzed building during the crossing of an HGV (heavy goods vehicle).

The analyzed building was designed about 100 years ago and the current traffic load conditions could not be taken into account. At the stage of construction of the building (in the 1920–30s), it was situated outside the town. As a result of territorial changes after World War II (the incorporation of rural areas into the city), construction of new industrial plants and changes in the transportation system, the heavy traffic load in the vicinity of the analyzed building increased. For obvious reasons, the building was not adapted to dynamic loads (because there were no such loads at that time), all the more seismic (the analyzed building is located in a non-seismic area). However, the main reason for the research was that residents of the building reported cracks/scratches in the walls of the building, which they linked to the increasing movement of heavy agricultural vehicles to a nearby transportation base. A conflict arose between the residents and the owners of the transportation base and the agricultural enterprise. Therefore, the conflict parties turned to an independent research unit with a request to establish the causes of cracks/scratches in the building and the impact of moving heavy vehicles on the building and its residents. At first glance, it might seem that the increased movement of heavy vehicles may have caused the appearance of cracks in the building. However, it had to be thoroughly analyzed taking into account vibration measurements, crack propagation in the building and estimation of traffic intensity.

**Figure 2:**
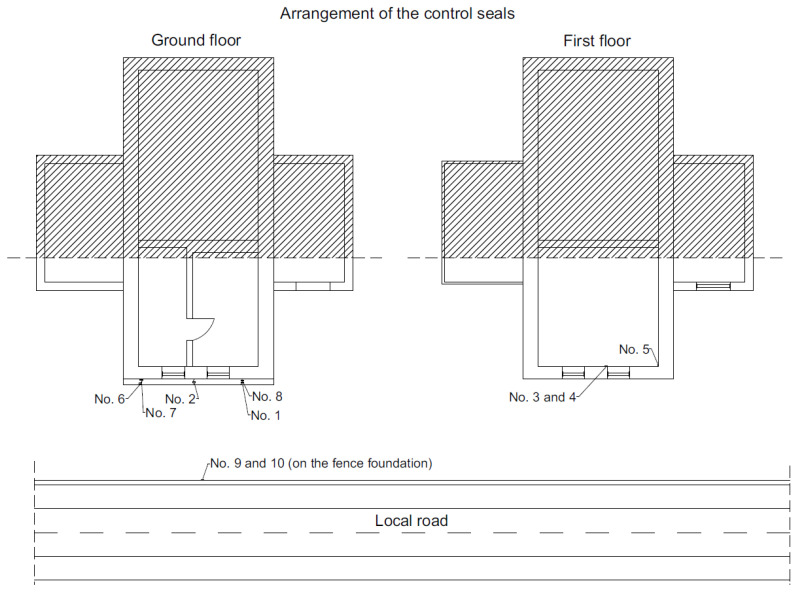
Arrangement of gypsum control tapes.

**Figure 3:**
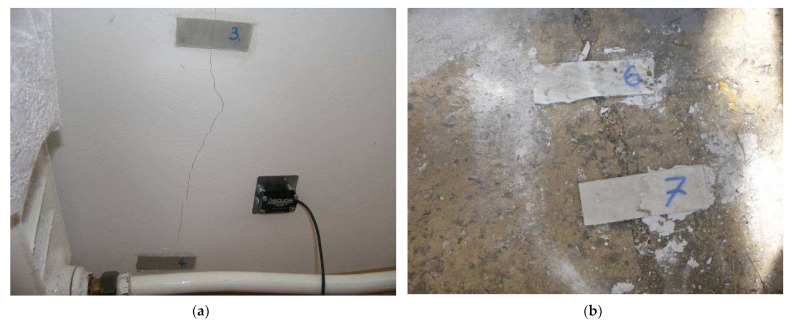
The view of control tapes: (**a**) No. 3 and 4 (inside the building—first floor) and (**b**) No. 6 and 7 on the concrete floor around the building.

To determine the impact of road traffic on the analyzed building, a detailed analysis of the damage was carried out. Figure 2 presents the arrangement of gypsum control tapes on localized cracks in the walls of the building (No. 1–8) and on the fence foundation near the road (No. 9–10). The purpose of the control tapes was to determine the potential of damage propagation. The gypsum control tapes were 80 × 30 mm in size and 5 mm in thickness. The control tapes were protected with a transparent glass plate. The visible cracks were classified as medium length and the control tapes were glued symmetrically at the 1/3 and 2/3 of the height of the cracks. The condition of the control tapes was monitored once a week for two months. This period was sufficient because during this period (two summer months) the heaviest vehicles (41.2 tons) passed via the analyzed road due to the harvest in the nearby fields. In other periods of the year, regular passenger cars only travel this road. Figure 3 presents the gypsum control tapes placed on the wall of the building.

During the monitoring of the condition of the control tapes and taking into account the general knowledge of brick structures, it was found that the damage in the analyzed building was of a non-structural character, i.e., cracks and scratches in plasters and coatings, loosening of the window and door fastenings in the walls. This damage was located on the first floor in the wall facing the road. Some cracks were also visible (not exceeding 1.5 mm) in the foundation of the fence and the concrete floor around the building. No crack propagation inside the building was noticed during the two-month monitoring of the control tapes. On the other hand, tapes attached to the outside of the building (concrete floor around the building, fence foundation) indicate a slight tendency to increase the cracks (no more than 2 mm). However, the authors do not know whether the foundation of the fence was reinforced.

**Figure 4:**
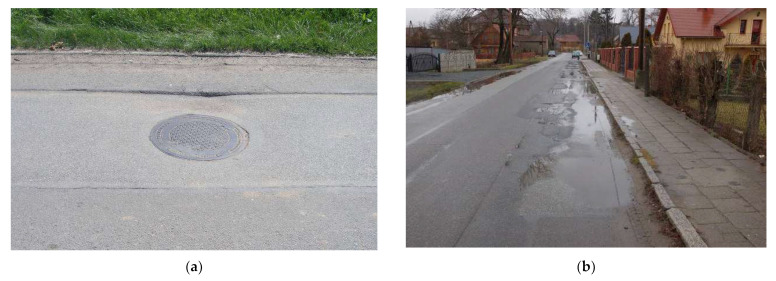
Examples of the roadway damage: (**a**) a collapsed well, (**b**) asphalt defects filled with water.

To determine the influence of road traffic on the analyzed building, the condition of the roadway near the building was assessed, which may increase the dynamic effects during the passage of vehicles. A lot of damage to the roadway (fractures and cavities) was identified. It was also observed that there were asphalt restorations (overlays) that caused unevenness of the roadway. Figure 4 presents the damage of the analyzed roadway.

At the time of inspection of the road, the following were found: (i) a number of cases of uneven surfaces all along the road, (ii) cavities on the surface, (iii) collapsed drainage and sewage wells, (iv) a lot of pollutants (sand, gravel) and vegetation near the pedestrian sidewalks that hindered drainage of the road, and (v) a lack of cross and longitudinal slopes of roadway (or being insufficient).

The roadway condition was assessed in accordance with appropriate guidelines and regulations [48,49] at the warning level (class C)—unsatisfactory condition, i.e., road with serious damages requiring repair.

It was found that the defects and unevenness of the roadway, as well as damage to manholes, might cause an increased dynamic effect. Therefore, additional crossings of HGV over the damaged areas have been planned.

### 2.2. Characteristics of the Road Traffic

Within a few days, full monitoring of the road traffic was carried out in order to understand its structure and assess its impact on the building and its inhabitants The greatest traffic of HGV was identified at around 8.00 (vehicles leave the transport base of a nearby company) and from 15.00 by 16:00 (vehicles return). It was observed that a total of 21 trucks passed on the analyzed road. Other agricultural equipment (tractors with attached trailers and agricultural machinery) was rare (2–4 per day). In most cases, passenger cars of the inhabitants of the surrounding villages used the road.

It should also be noted that the road has a specific position, i.e., it is relatively short (180 m) and ends with sharp turns causing a significant slowdown in vehicle speed. As a result, vehicles using this road usually do not exceed the speed of 30 km/h.

Full traffic observations were performed twice. The categorization of vehicles was made in accordance with guidelines from [48]. The observers used the method of direct manual survey (the measuring points were located in the analyzed building). The observed road was classified as a “W” type according to [48], i.e., the section on which the direct traffic measurement takes place over a specified number of hours. Therefore, in this case, the measurement time was limited to 8 h (8:00–16:00) on each measurement day.

As a result of measuring the traffic structure on the observed road, it was noted that:

- Most of the time, traffic is light—almost 97% of all traffic and takes place mainly between 11.00 and 15.00;
- There is almost never a lot of traffic, i.e., 8–10 passages, mainly from 7.00 to 8.00 and from 15.00 to 16.00;
- All-day monitoring (8.00–16.00) showed 202 vehicle passages.

### 2.3. Measurement Methodology

According to the Standard [50], building vibrations should be determined by displacements (or velocity or acceleration) in characteristic points of the building and at the appropriate vibration frequency. Measuring vibration acceleration as a function of frequency was carried out to determine the impact of vehicles moving along the observed road on the analyzed building. Three crossings were included in the measurement:

- A car with a mass of 2.0 t passing at 20 and 40 km/h;
- A combine harvester with a mass of 13 t traveling at a speed of 15 km/h (maximum speed);
- HGV 41.2 t traveling at a speed of 35–40 km/h.

In fact, only a few trucks, most of them unloaded (<20 t in weight), pass each day.

The experimental tests used a truck loaded with corn (41.2 tons total weight) traveling at a speed of 35–40 km/h (more than usual). During the tests, members of the research team informed the driver about the possibility of safe driving at speeds higher than allowed.

For short-term vibrations, i.e., the total duration of which does not exceed 3 min/24 h (as in the analyzed case), the selected parameter was recorded in at least three load cycles, i.e., the selected vehicles ran at least three times for each measuring point. The *x* and *y* designations for the horizontal directions of measurement and *z* for the vertical directions were adopted according to the Cartesian coordinate system. In the analyzed case, the *x* and *y* directions were on the wall from the vibration source, at the level of the surrounding soil. The recorded signals were analyzed in the vibration range from 0.5 to 80 Hz [50].

**Figure 5:**
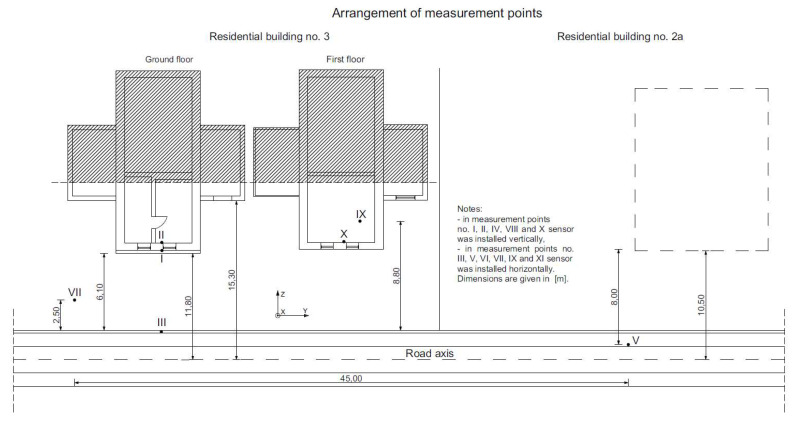
Arrangement of measuring points.

**Figure 6:**
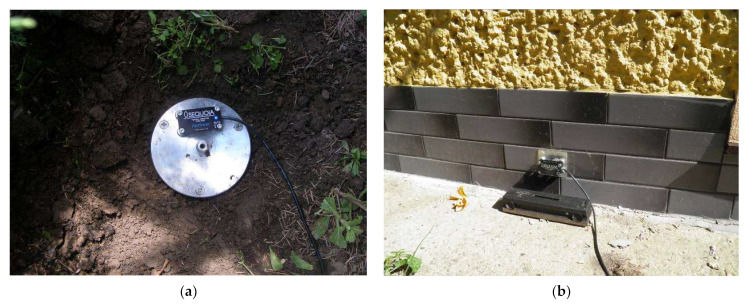
View on measuring points: (**a**) No. VII in soil, (**b**) No. I—situated on the outer wall of the building.

The field tests were conducted at the following measuring points (Figure 5):

(a) Inside the building:
  - On the load-bearing wall at ground level (on the external facade of the building—Figure 6a) measuring point No. I;
  - On the load-bearing wall at ground level (inside the building, from the side of the vibration source)—measuring points No. II and VIII;
  - On the first floor, on the load-bearing wall (measuring point No. X) and on the floor (measuring point No. IX)—research related to the determination of the impact of vibrations on humans in the building.
(b) Outside the building, along the wave path from the vibration source to the building):
  - On the foundation of the fence (measuring points No. III and IV);
  - On the pavement (measuring point No. V);
  - On a special tripod situated in the soil (measuring points No. VI, VII and XI)—Figure 6b.

The FastTracer vibration gauge by Sequoia was applied in the tests. It enables the measurement of vibrations simultaneously in three independent directions. Technical data of the gauge are: (i) frequency range 0–2500 Hz, (ii) maximum acceleration +/− 5 g, (iii) sampling rate 8192 samples/s, (iv) resolution 0.0025 m/s^2^, and (v) noise 0.075 m/s^2^. The Signal was saved as a text file then transformed into a .mat file and processed in Matlab.

Each measuring point was cleaned before mounting the gauge. The gauge was permanently fixed with four neodymium magnets to a steel sheet, which was then attached to the measuring point with double-sided tape. This mounting method has been verified in other experiments [46].

**Figure 7:**
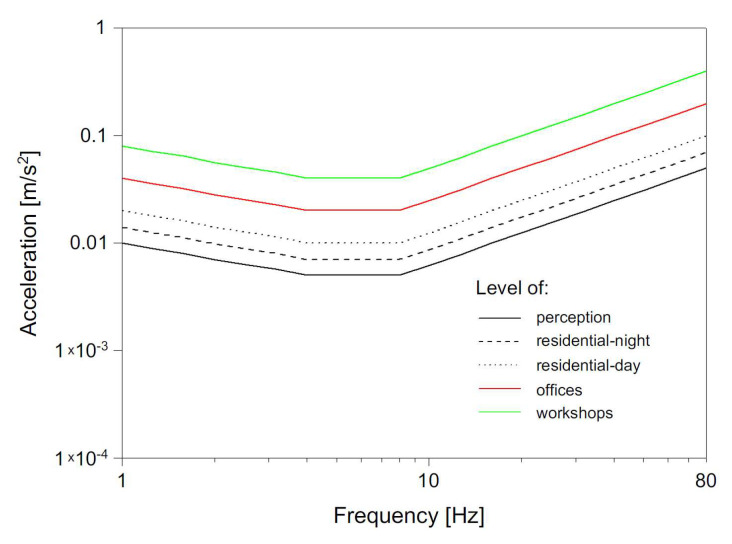
Perception level for vertical RMS acceleration and its multiplication for various conditions according to international standards [50].

The analysis involved time history analysis, an FFT (Fast Fourier Transform) analysis as well as velocity and RMS (Root Mean Square) acceleration in a one-third octave bands spectrum. Previously, the methodology of velocity analysis was used for railway-induced vibration [11,12]. Study [46] has shown that this method can be implemented for other vibration sources but has not yet been verified for road-induced vibration. Therefore, in this study, it was decided to use the above-mentioned method. Velocity and RMS acceleration in the one-third octave bands spectrum were compared to reference levels: perception level and annoyance level for residential buildings at night and day, and offices and workshops. Annoyance levels were acquired by multiplication of perception level by specified factor *n* = 1.4 for residential buildings at night and *n* = 4 for residential buildings at day (Table 1), according to the literature [51,52]. Annoyance levels for various conditions are presented in Figure 7, in the case of vertical acceleration levels. In Section 3 (Measurement Results and Discussion), three factors of *n* = 1, *n* = 1.4 and *n* = 2 (Table 1), were applied to analyze vibration records (Figures 10 and 11).

**Figure 8:**
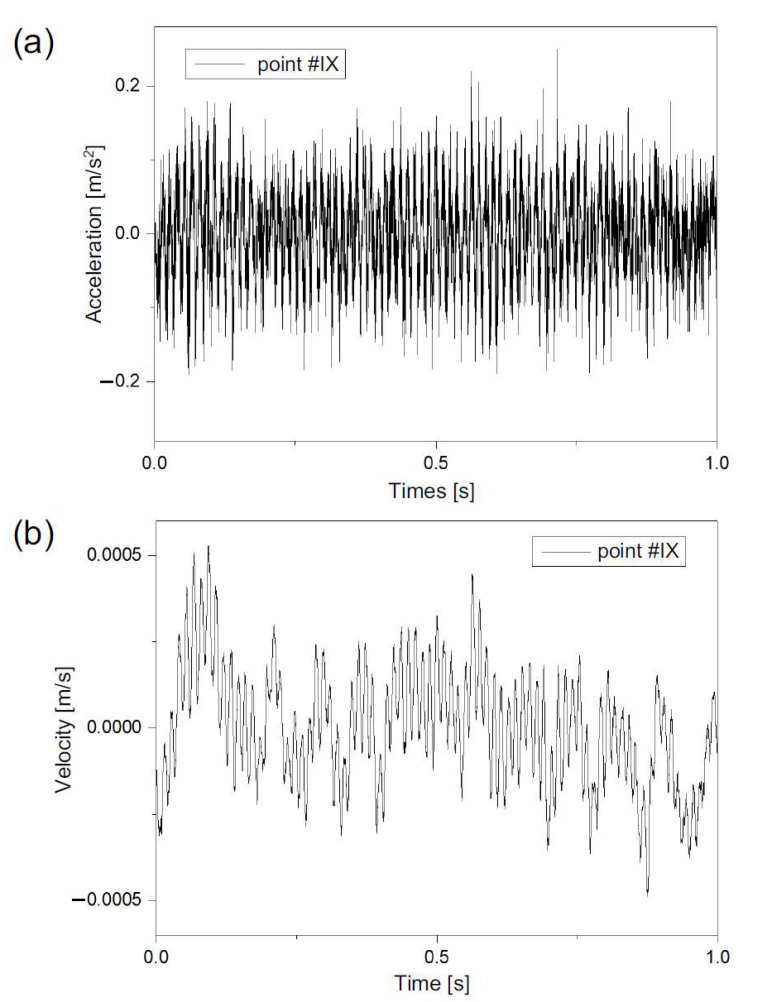
Transformation from acceleration (**a**) to velocity (**b**) for point No. IX in the *z* direction.

**Figure 9:**
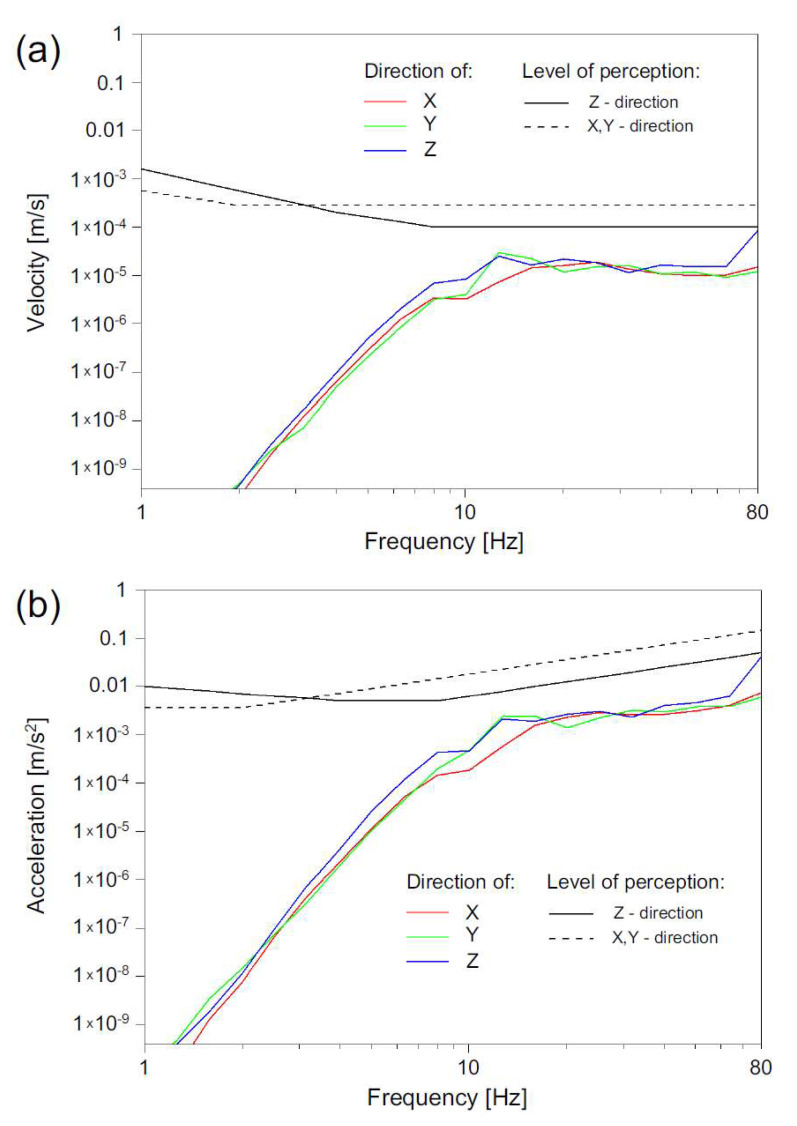
Comparison of velocity (**a**) and acceleration (**b**) levels to perception levels for horizontal (*x*, *y*) and vertical (*z*) directions of point No. IX.

Vibration records for seven points were selected for the analysis. Points No. I, II, III, V, VII, IX and X were presented in the scheme in Figure 5. Points No. III, V and VII were localized on the ground at a close distance to the road, while points No. I, II, IX and X were localized in the building (I and II on the first floor, and IX and X on the second floor). Vibrations were measured in three directions *x*, *y* and *z*. The *x* direction was horizontal, transverse (perpendicular) to the road axis, the *y* direction was horizontal lateral to the road axis, and the *z* direction was vertical. Vibration analysis was carried out with the use of acceleration records and for calculated velocities. Velocities were calculated with the use of the inverse Fast Fourier Transform (iFFT) method, which is one of the most precise methods for numerical derivatives [53]. After time history analysis, the signal was filtered with the use of a series of band-pass filters. The 20 one-third octave bands with central frequencies from 1 to 80 Hz were applied. For each band, the RMS value was calculated. The band’s boundaries were designed with the reference to ISO 2631-2: 2003 [50] and PN-B-02171: 2017 [54]. Transformation of acceleration to velocity results for the selected point and direction is presented in Figure 8. An example of RMS analysis for a selected point is presented in Figure 9 where the velocity and acceleration in three directions *x*, *y* and *z*, were compared to perception levels accordingly.

**Table 1:** Multiplication factors of the perception level for various conditions, on the base on international standards [50]. In bold are factors used in the presented research.

Building Type	Time of Day	Multiplication Factor *n*			
		Continuous Vibration [54]	Accidental Vibration [54]	Continuous Vibration [51,52]	Accidental Vibration [51,52]
Hospitals (operating room)	night day	**1**	1	1	1
Hospitals (sickroom)	night	1	4	1	1
	day	2	8		
Residential buildings	night	**1.4**	4	1.4	1.4–20
	day	**4**	32	**2**–4	30–90
offices	night day	**4**	64	4	60–128
workshops	night day	**8**	128	8	90–128

## 3. Measurement Results and Discussion

### 3.1. Initial Remarks

An extra measurement of vibration accelerations inside the analyzed building was carried out to set the harmfulness of vibrations associated with the traffic on the road for humans, according to [50]. The vibration velocities inside the building were calculated based on the integration of the accelerations received from the field tests.

The experimental investigations were carried out in places where humans commonly stayed (on the first floor) and at the points where vibrations were transmitted from the construction to humans (on the floor—measuring point No. IX and on the building wall from the source of vibration—measuring point No. X—Figure 5). Because a human can perceive vibrations in any direction, tests were also executed in three directions: two horizontal ones (*x* and *y*) and vertical (*z*) signed with a eutectic system associated with the geometry of the measuring site. The vibrations were recorded while driving a truck weighing 41.2 tons at a speed of 35–40 km/h.

The field tests were conducted in the most unfavorable conditions in the entire assessed frequency band in terms of the impact on humans. According to the standard, the recorded vibrations were classified as discontinuous, i.e., with a total duration not exceeding 30 min in a 24 h period.

Taking into account the damage observations and measurement results obtained, it was found that the damage to the building was not caused by nearby traffic. Additionally, it is possible to exclude other sources of damage, e.g., settlement of foundations, as they occur at the beginning of the building’s operation (in this case they were stabilized). It was also noticed that the residential building was operated as intended (no source of vibration in the building). There were also no modifications (or changes) to the building structure that could cause scratches/cracks. Therefore, most likely, the occurring damage was caused by the normal operation of the building and the wear of materials, especially since the occurring cracks were not of a propagating nature.

### 3.2. Velocity Analysis

It should be underlined that there were no velocity-sensitive devices in the building; nevertheless, velocity analysis is important for estimating the impact of the road traffic on the building and its residents.

**Figure 10:**
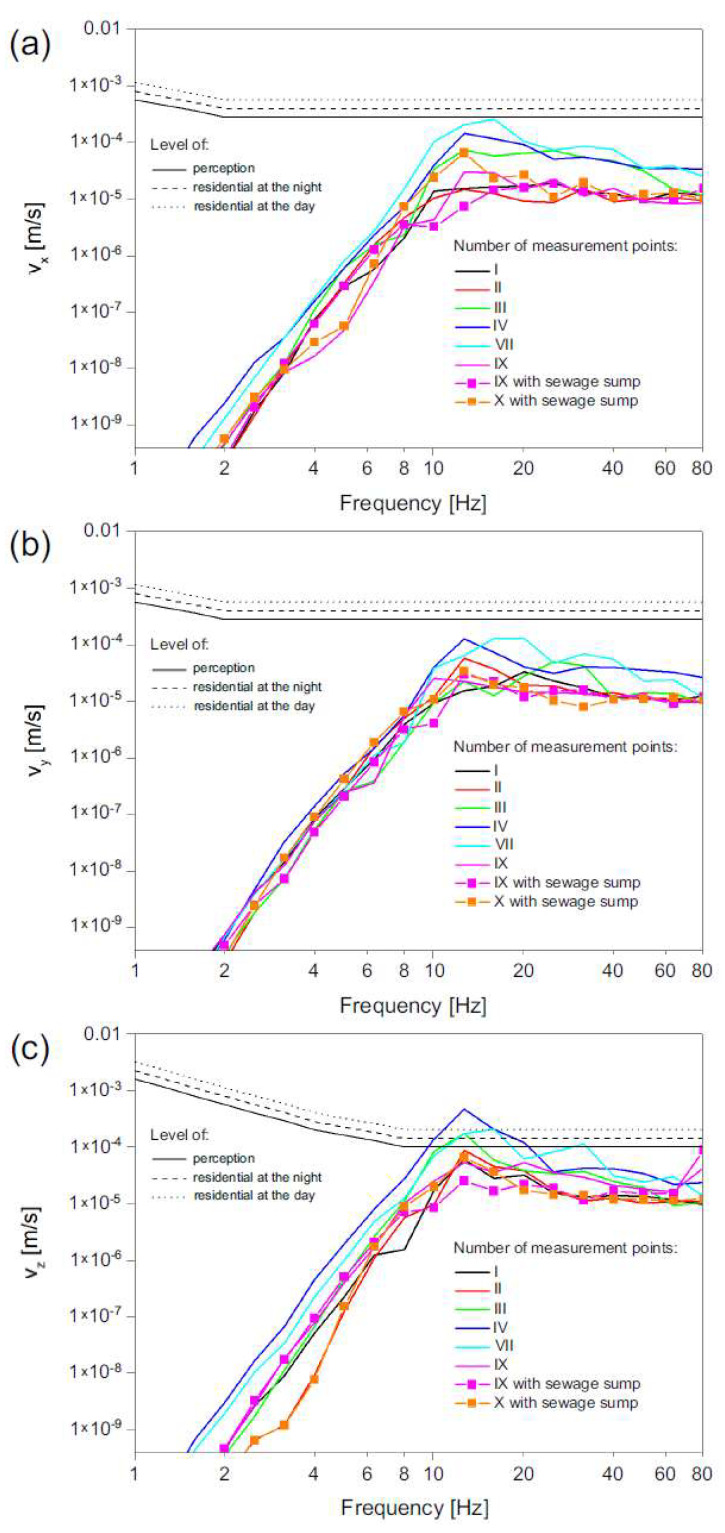
RMS velocity level (speed of 40 km/h) at selected points in: (**a**) *x* direction (transverse to the road axis), (**b**) *y* direction (lateral to the road axis), (**c**) *z* direction (vertical velocity).

In the vertical direction, perception and annoyance levels were contravened for frequencies between 10 and 20 Hz in the case of measured points localized on the ground at a close distance to the road—points No. III, V and VII. The strongest contravention was observed at point No. V, at a frequency of 12.5 Hz (Figure 10c).

One can note that only in the vertical direction “*z*” were the perception and annoyance levels contravened (Figure 10c). They were recorded at three points No. III, V and VII (Figure 10), i.e., located in the immediate vicinity of the road. Analyzing the obtained results, one can observe that as the distance from the road increases, the amplitudes of the vibrations decrease (points No. I and II), while the response of the building causes an increased amplitude of the vibrations measured on the first floor of the building (points No. IX and X). Therefore, such an effect was caused by the damping of the ground and obstacles between the source and the measured structure (a fence made of a massive concrete foundation). As observed, this foundation acts as an insulator that causes damping of the vibrations induced by passing vehicles. At the same time, it is not possible to quantitatively estimate the effect of the fence foundation on vibration damping as well as the effect of ground damping. By analyzing all the directions and points for which the measurements were carried out, it can be noted that the vertical vibration has a much greater amplitude than the horizontal one. In addition, horizontal vibrations in the direction perpendicular to the road have greater amplitudes than those in the direction parallel to the road.

The results indicate that there was no contravention of perception level for both horizontal directions for any of the analyzed passages (Figure 10a,b). Vibration levels in points localized in the building on both the first and second floor, points No. I, II, IX and X, were lower than the perception level in all three directions. In point No. VII, localized on the ground beside the road, the perception level was hardly reached in the *x* direction.

### 3.3. Acceleration Analysis

**Figure 11:**
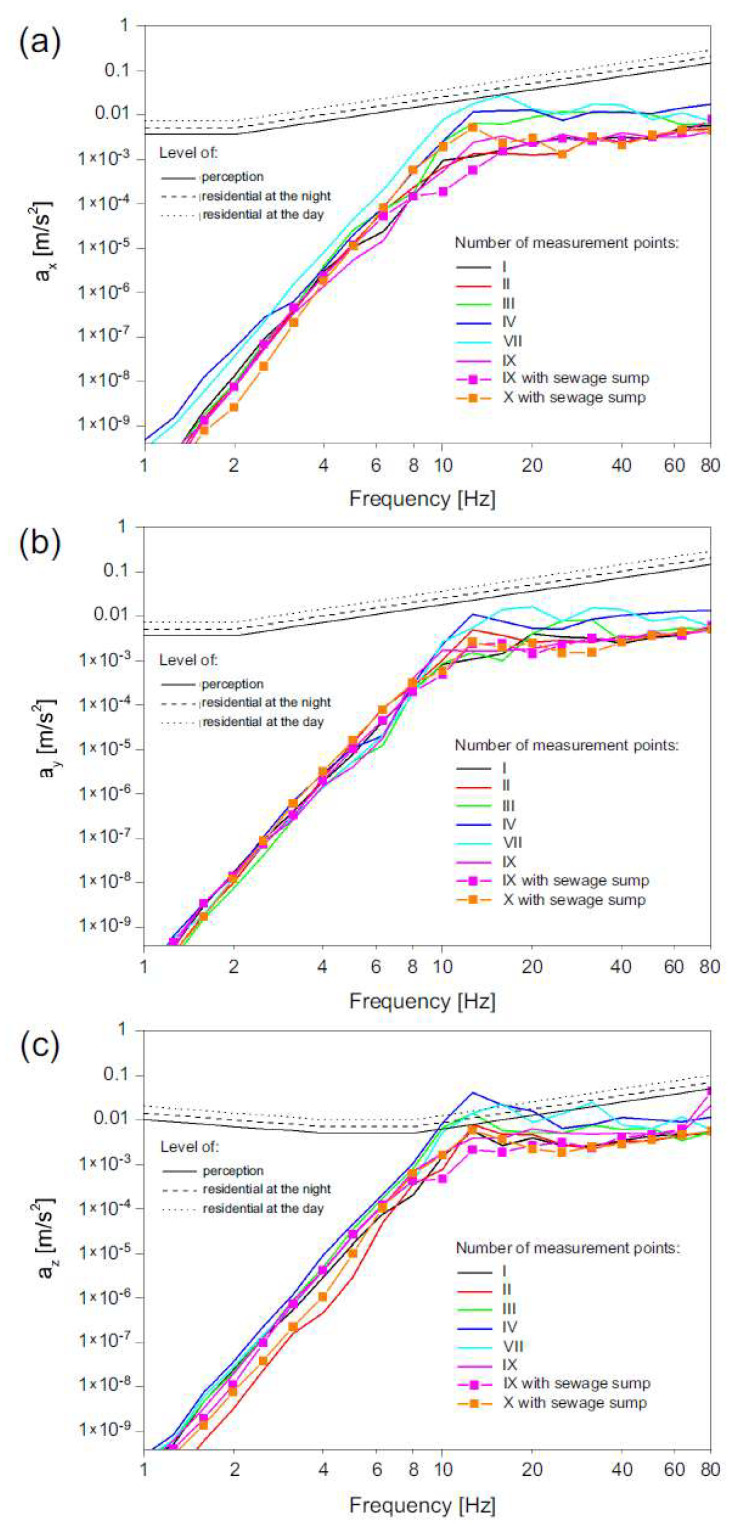
RMS acceleration level (speed of 40 km/h) at selected points in: (**a**) *x* direction (transverse to the road axis), (**b**) *y* direction (lateral to the road axis), (**c**) *z* direction (vertical acceleration).

Observation of RMS acceleration indicates that there was no contravention of perception level for both horizontal directions for any of the analyzed passages (Figure 11a,b). In the vertical direction, perception levels and annoyance levels were contravened for frequencies between 10 and 20 Hz in the case of measured points localized on the ground at a close distance to the road—points No. III, V and VII (Figure 11c). Vibration levels at points No. I, II, IX and X, localized in the building on both the first and second floors were lower than perception levels in all three directions.

Moreover, the vibration analysis at the acceleration level has the same qualitative effect as the velocity level analysis, and thus no difference was observed in the case of the vibration perception level at the measured points for the velocity and acceleration analysis.

### 3.4. Frequency Range of Vibrations

A contravention of the perception level in the vertical direction was observed only in points localized on the ground, at a close distance to the road, 3–8 m to the road axis. The dominating frequencies between 10 and 20 Hz were likely to be related to local ground conditions. In points localized in the building, the vibration level was lower, since the building was localized at a bigger distance to the road axis, ca. 12 m, and vibrations were attenuated.

Vertical and horizontal vibration levels were of a very similar range; however, insignificantly higher maximum values were observed in the vertical direction for both acceleration and velocity observations in points localized on the ground (points No. III, V and VII). In points localized in the building (points No. I, II, IX and X) vertical and horizontal vibration levels were of a very similar range. Contravention of perception levels only in the vertical direction was additionally caused by lower perception levels for the vertical direction. The difference in perception level for the vertical and horizontal directions was the consequence of the fact that the human body is more likely to percept vertical rather than horizontal vibrations in frequency ranges between 4 and 80 Hz [46,51,52].

It should be emphasized that the passage of the car through the sewage sump (points No. IX and X) did not cause a contravention of the permissible levels of the observed vibrations in residential buildings. The obtained acceleration and velocity values from these points did not reach the maximum values. They were lower than observed at points No. III, V and VII, where the maximum values contravening the permissible values were obtained.

## 4. Conclusions

Based on the experimental analysis, it can be pointed out that the obtained velocity values in the tested building induced by the passage of various vehicles were below the permissible levels [50]. However, it was noticed that the distance between the building and the fence (including the concrete foundation) plays an important role because it dampens the vibrations induced by passing vehicles. Therefore, the construction of a fence together with a concrete foundation can be a positive impact on the comfort of residents of buildings exposed to vibrations from passing vehicles. In addition, the following specific conclusions can be drawn:

- Despite the short distance of the building from the vibration source, the recorded values do not exceed the thresholds of perception in an old residential building.
- Vibrations recorded on the first floor have higher amplitudes compared to vibrations recorded on the ground floor. The reason was the increased amplitude of vibrations caused by the building’s response to excitation (traffic vibration). Vibrations caused by vehicles can be observed in the range of 8–80 Hz.
- Passage of the vehicle through the sewage sump does not increase the amplitude of the measured vibrations in the range from 1 to 60 Hz, while a noticeable amplitude increase occurs at higher frequencies.
- Vibration analysis in terms of acceleration and velocity gives the same qualitative effect; no difference was observed in contravention of the thresholds of perception at the measured points between the velocity and acceleration analysis. It is not necessary to use sensors that measure acceleration at low frequencies of 1–8 Hz.
- Additionally, it should be emphasized that vibration analysis in the aspect of annoyance for inhabitants should be carried out together with noise analysis, since it is not obvious which factor causes stronger psychophysical annoyance: vibration or noise. Vibration analysis is carried out in the frequency range of 1–80 Hz, and noise is examined in the frequency range of 16–20,000 Hz. Annoyance can be caused by vibration at higher frequencies than 80 Hz.
- The damage observed in the analyzed building was classified as non-structural, i.e., cracks and scratches in coatings and plasters. The crack propagation monitoring carried out over the period of two months did not show that the damage has a tendency to develop further. Thus, the cracks could be caused by other factors, e.g., normal exploitation and wear of materials.
- The use of analysis in a one-third octave bands spectrum for both acceleration and velocity gives additional possibilities in determining vibrations burdensome for humans.
- The condition of the road pavement was classified at the warning level in accordance with [48] (class C—unsatisfactory condition). There were numerous voids in the road pavement, surface unevenness, damaged sewage manholes and contamination in the vicinity of the sidewalk.
- It is recommended to repair the road, with particular emphasis on drainage and sewage manholes as well as defects in the asphalt surface, and the modeling of appropriate transverse and longitudinal slopes. In the light of the conducted research and analyses, it is not recommended to limit the load of vehicles moving on the considered road.

Thus, according to the research, the problem of the vibration effect on people is important and had been raised by scientists, especially, currently, regarding the aspect of sustainable infrastructure development. This is a complex problem and should always be considered when planning the construction of new roads and industrial plants close to human habitats. The presented case study may be of use to other researchers who will be involved in similar cases. Further research will be extended to include additional types of loads with different road surfaces and different vehicle speeds, as well as additional analyses including noise level tests.

## Data Availability

The data presented in this study are available within the article.
